# Utilizing novel TBI-on-a-chip device to link physical impacts to neurodegeneration and decipher primary and secondary injury mechanisms

**DOI:** 10.1038/s41598-022-14937-w

**Published:** 2022-07-12

**Authors:** Edmond A. Rogers, Timothy Beauclair, Andrew Thyen, Riyi Shi

**Affiliations:** 1 Weldon School of Biomedical Engineering, Purdue University, West Lafayette, IN 47907 USA; 2 Department of Basic Medical Sciences, School of Veterinary Medicine, Purdue University, West Lafayette, IN 47907 USA; 3 Center for Paralysis Research, Purdue University, West Lafayette, IN 47907 USA; 4 Indiana University School of Medicine, Indianapolis, IN 46033 USA

**Keywords:** Biochemistry, Neuroscience, Pathogenesis

## Abstract

While clinical observations have confirmed a link between the development of neurodegenerative diseases and traumatic brain injuries (TBI), there are currently no treatments available and the underlying mechanisms remain elusive. In response, we have developed an in vitro pendulum trauma model capable of imparting rapid acceleration injuries to neuronal networks grown on microelectrode arrays within a clinically relevant range of g forces, with real-time electrophysiological and morphological monitoring. By coupling a primary physical insult with the quantification of post-impact levels of known biochemical pathological markers, we demonstrate the capability of our system to delineate and investigate the primary and secondary injury mechanisms leading to post-impact neurodegeneration. Specifically, impact experiments reveal significant, force-dependent increases in the pro-inflammatory, oxidative stress marker acrolein at 24 h post-impact. The elevation of acrolein was augmented by escalating g force exposures (30–200 g), increasing the number of rapidly repeated impacts (4–6 s interval, 3, 5 and 10×), and by exposing impacted cells to 40 mM ethanol, a known comorbidity of TBI. The elevated levels of acrolein following multiple impacts could be reduced by increasing time-intervals between repeated hits. In addition, we show that conditioned media from maximally-impacted cultures can cause cellular acrolein elevation when introduced to non-impact, control networks, further solidifying acrolein’s role as a diffusive-factor in post-TBI secondary injuries. Finally, morphological data reveals post-impact acrolein generation to be primarily confined to soma, with some emergence in cellular processes. In conclusion, this novel technology provides accurate, physical insults with a unique level of structural and temporal resolution, facilitating the investigation of post-TBI neurodegeneration.

## Introduction

Traumatic Brain Injury (TBI) is the leading cause of death for Americans between 1 and 44 years of age^1^, with an alarming increase in the number of military personnel exposed to blasts and other head injuries^2–4^. These statistics represent an approximately 5.3 million Americans who are currently living with a TBI-related disability, producing a staggering economic burden in excess of $76.5 billion^5^. While these enormous social and economic costs of TBI are clear, the underlying mechanisms are still not well understood. Consequently, despite elevated social awareness and the ever-increasing incidence of TBI among people from all backgrounds, few (if any) treatments are currently available: over 30 large clinical trials have failed to distinguish a treatment capable of producing a dependable and measurable difference in TBI patients^6^. To address this complex, presently unmet medical challenge, our understanding of the associated pathogenic mechanisms must be enhanced, ultimately facilitating earlier disease detection and more effective therapeutic interventions, to not only benefit patients but also their families and society as a whole.

In order to better understand the pathogenesis of TBI, a suitable and effective experimental injury model is critical. To date, most brain trauma models have been primarily focused on in vivo approaches, which have played a crucial role in improving our understanding of the pathological process and uncovering key biomarkers. However, these procedures are ethically and procedurally complex, and generally lack the necessary level of resolution required for mechanistic investigations on a cellular level. Therefore, an in vitro approach, providing subcellular access and more precise control of experimental conditions, is paramount for dissecting molecular pathways of trauma-induced pathologies, as recent reviews suggest^7, 8^. Such in vitro investigations can be combined with animal models to not only recapitulate clinically relevant injury scenarios, but also reveal meaningful insights for higher complexity levels.

It is well understood that mechanically-induced neurotrauma (i.e. TBI) consists of both primary (physical) and secondary (chemical) injury components^9–14^. While primary injuries are sudden and somewhat obvious, secondary injuries are longer lasting, far more complex, and less discernable^15^. These destructive, self-propagating changes, commonly referred to as “secondary injuries”, occur gradually (hours to weeks) after the initial primary mechanical insult, are usually mediated through chemical factors, and ultimately result in tissue dysfunction or cell death^16–18^. Further, a link between these secondary chemical injuries and long-term post-TBI neurological damage has been demonstrated^19^. In fact, in mild TBI, the most commonly occurring type of TBI^20^, induced secondary injuries are suggested to be the dominant pathology leading to post-TBI neurological sequela (i.e. Alzheimer’s Disease (AD) and Parkinson’s Disease (PD))^21–23^. The current investigation focused on a relatively acute (24 h) phase of secondary injury, which, should ultimately be expanded to provide a more complete evaluation. Furthermore, based on previous studies it is evident that these post-injury events, including those initiated during the earlier phases following the initial trauma, may have a significant effect on not only acute, but also chronic injury (i.e. oxidative stress). As such, the acute stage of secondary injury (24 h) is of great importance and was the focus of this investigation^11, 12, 16–18, 24^. Despite its significance, secondary injury pathways and the underlying mechanisms are not well defined, likely due in part to the limitations of current experimental models. An understanding of these secondary injury-instigated mechanisms of pathogenesis is critical for our efforts to alleviate post-TBI complications, and will require an in vitro system where such detailed investigations are possible and mechanisms can be deconstructed for not only potential treatments, but also diagnosis.

To this end, we intended to investigate the mechanisms of secondary injury using an in vitro system developed by our group, referred to as “TBI-on-a-chip”. We have previously shown the functional relevance of this model by demonstrating force-dependent pathological changes in electrical activity with neuronal networks grown on microelectrode arrays^25^. In the current study, we examine delayed, biochemical changes in response to clinically relevant mechanical concussive impact injuries. In addition, this in vitro model also allows for the isolation of endogenous secondary injuries from primary, mechanical insults.

Acrolein is an αβ-unsaturated aldehyde and demonstrated trauma biomarker^13, 14, 26^ whose increase post-injury has been demonstrated in multiple models of trauma and TBI, and has already been established as a likely component of secondary injury mechanisms^12, 13, 24, 27^. It is one of several markers of oxidative stress produced in the brain post-TBI, and, coupled with a trauma-induced decrease of antioxidant defense enzymes, creates an imbalance that is not only directly related to the pathogenesis of TBI, but also an attractive target for intervention strategies^28–30^. However, these post-neurotrauma acrolein-mediated pathologies have been primarily investigated in live animal injury models. Due to the limitations of in vivo preparations, a detailed time course of elevation, particularly in the early/acute post-injury stage, has not been investigated. As such, we wanted to utilize this model to study the detailed evolution of this pathological mechanism, including the dynamics of secondary injury. This includes intracellular, temporal, and spatial features of acrolein activity, as well as other relevant parameters related to its role in secondary injury mechanisms following single, intermittent, and consecutive-repeated injuries. In addition, we capitalize on the model’s precise command of physiological parameters to demonstrate its capability for investigating relevant comorbidities of TBI, such as co-exposure to alcohol, with relative ease.

Oxidative stress is a hallmark of mechanical trauma in the CNS, and acrolein is a well-known marker for this pathology^14, 31–33^. While physical impact induced CNS trauma has been shown to produce multiple oxidative stress-related chemicals that could potentially be used as an indicator of secondary injury^10, 34, 35^, we selected acrolein based on the following criteria. First, acrolein has been well-established as a key-player in secondary injury mechanisms following mechanical impact using various animal models of CNS trauma^12–14, 18, 24, 27^. Further, these studies have also demonstrated that elevated acrolein levels post-trauma are capable of eliciting a slew of pathological neuronal changes^14^. Acrolein damages neuronal tissues by poisoning mitochondria, compromising the integrity of neuronal membranes, and degrading myelin^33, 36–42^. This has been shown not only in rodents, but also in canine following CNS trauma^43^, and even in human’s with chronic neurodegenerative diseases where oxidative stress and inflammation are known causalities, such as multiple sclerosis^44^. Therefore, determining if this acrolein pathology could be recapitulated with our in vitro system could be a critical step towards strengthening the model’s correlation with in vivo studies, and ultimately its clinical-relevance. Second, acrolein is formed in at least 40× greater concentrations and is 100× more reactive than other aldehydes^45–47^. As both a product of, and catalyst for lipid peroxidation, acrolein induces a vicious cycle of oxidative stress, significantly amplifying the effects and continuously propagating degeneration in CNS trauma^14, 18, 48^. Therefore, acrolein has been shown to cause disproportionately higher neuronal damage when compared to other aldehydes, making it a suitable target-compound for observation and providing a representative summary of overall aldehyde toxicity^14, 26^. Furthermore, it has been demonstrated that the reduction of acrolein via aldehyde scavengers can offer significant neuroprotection^32, 49–52^, confirming acrolein’s role as not only a representative marker of secondary injury, but also a key indicator for diagnosis, and a viable target for future investigations into potential therapeutics and pharmaceutical treatments.

In the current study, utilizing morphological analysis and this TBI-on-a-chip model, we report significant, impact-force and quantity dependent increases of cellular acrolein-Lys adducts at 24 h post-injury. In addition, we also note that media from impacted cells is capable of significantly escalating intracellular acrolein levels in unimpacted networks, further strengthening acrolein’s role as a diffusive factor in secondary injury. Last, we show that acrolein is further significantly raised when clinically relevant alcohol concentrations were co-applied with impacts, indicating a synergistic pathological effect from a known TBI comorbidity.

## Methods

### Primary neuron culture

Frontal cortex tissues dissociated from E-16 embryos of ICR mice (Envigo, Inc) were cultured based on the pioneering protocols of Ransom et al.^53^ with minor modifications^54^. Cortices were mechanically separated, enzymatically digested with a 0.05% trypsin solution (Gibco), triturated, combined with Dulbecco’s Modified Minimal Essential Medium (DMEM), supplemented with 4% fetal bovine serum (Gibco) and 5% horse serum (Gibco), and seeded at 50–70 k cells per 100 µL onto 5 × 5 cm microelectrode arrays (MEAs) (~ 3 mm diameter adhesion island; ~ 7 mm^2^ area), yielding approximately 300 neurons per mm^2^ (~ 2100 total) after network formation on a carpet of glial cells. The use of embryonic tissue for network generation provides a mixture of neuronal and glial cell types that are representative of the parent tissue^55^. Cultures were subsequently maintained at 37 °C in a 10% CO_2_ atmosphere and transitioned into a medium containing 5% horse serum 2 days post-seeding, followed by biweekly medium changes. Experiments and animal care were performed in compliance with the Purdue University Animal Care and Use Committee’s guidelines, under institutional protocol 1306999879, as well as the recommendations in the ARRIVE guidelines.

### Injury model

**Figure 1:**
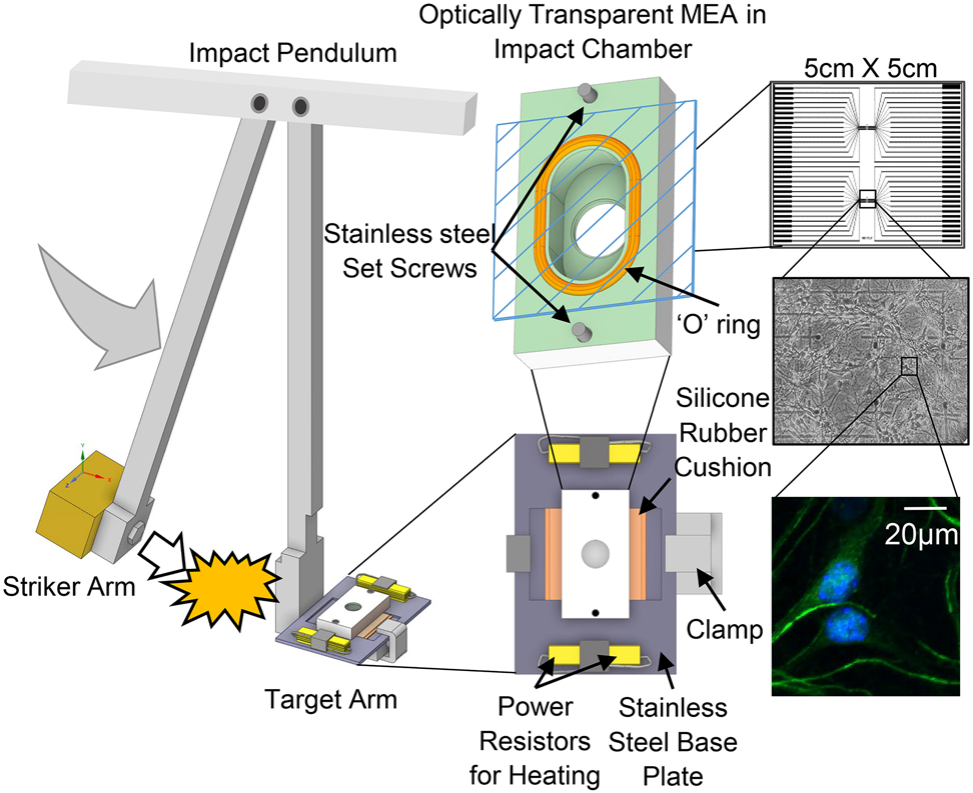
“TBI-on-a-chip” Injury model. Acceleration injury is generated by the impact pendulum (left): the striker arm swings down from a preselected height, making contact with the target arm and stainless steel baseplate/chamber assembly (enlarged). This assembly is airtight and functions as a sort of “miniature-incubator,” capable of protecting the network adhered to MEA while maintaining 5 mL of culture media at physiological pH, osmolarity, and temperature, for the duration of the impact application. The base plate includes 2 pairs of 4 Ω resistors for heat generation, and a silicone rubber cushion for the MEA. Two stainless steel set screws connect the chamber block to the base plate. Inverting the chamber reveals a silicone “O” ring, which connects to the topside of the optically transparent MEA (represented with cross hatching, and enlarged on right). The microscope window (70 µm cover glass) provides morphological access to networks. A typical MEA conductor pattern (two groups of 32 transparent indium tin oxide cruciform electrodes, spin coated with methyl-trimethoxysilane resin, laser deinsulated and electrolytically gold plated) is shown, with and without cells (right). An enlarged fluorescent image illustrates the type of resolution available with this system.

The in vitro pendulum model was selected for its ability to apply uniform, tangential acceleration injuries to networks while minimizing secondary physical-influences with limited cell death, as previously described^25^ and briefly summarized in Fig. 1. Mature networks on MEAs were aseptically transferred to custom, stainless-steel experimental chambers capable of maintaining homeostatic conditions while withstanding high g forces (up to 300 g), and securely attached to the impact pendulum (target arm). The weighted arm of the pendulum swings down (striker arm) from a preselected height and g force level, making contact with the target arm and network-chamber assembly. The result is consistent applications of force in a system that can be modified to accommodate a range of force-distributions by adjusting both the accelerations and contact times, two critical components of impact injuries^56^. Although this morphological study did not include electrophysiological recordings, we used MEAs for consistency of the adhesion and life support environment. However, when our optically transparent MEAs fabricated in-house are combined with a Multichannel Acquisition Processor System from Plexon Inc. (Dallas, Tx.), this novel in vitro impact system provides electrophysiological recordings with sub-cellular morphological access in real-time. A portion of a typical recording matrix used (MMEP-5, with two separate recording islands of 32 electrodes each, 64 electrodes total, and cruciform electrode design) is shown in Fig. 1 with a corresponding fluorescent image taken from a network at 7 days in vitro. This design provides two separate, but age and maintenance-matched networks per chip. While these recording capabilities are impressive and the center point of a previous publication^25^, they were not the focus of this investigation.

Each experimental treatment was performed concurrently with procedurally and age matched control networks. Thus experimental and control network handling was identical, with the notable exception of the application of force (no impact) for control networks. This included the simultaneous removal from incubator and placement into separate, non-impacted chamber-assemblies, with “mock-impact” attachment to the pendulum target arm, and subsequent return to incubator. Culture storage in incubator requires the sterile assembly/disassembly of a biologically inert silicone gasket (volume 3 mL), sealed with silicone grease, with insertion into impact chamber. Each culture for all treatments was fixed at 24 h post final injury application.

### Injury protocols and chemical treatments

#### Single and multiple impacts

Single impacts of 30, 100, or 200 g were applied, and endogenous acrolein (acrolein lysine adducts, or acrolein-Lys adducts) production was quantified at 24 h post-impact. Subsequently, impact-multiples of 30 and 200 g were rapidly (4–6 s between hits) administered in series of three, five, and ten times, also with the acrolein levels quantified at 24 h post final impact. Acceleration forces, or “g” as commonly described in TBI-literature, were determined through a series of experiments utilizing multiple accelerometer placements^25^. The values 30, 100, and 200 g were particularly selected for their distribution through the range of clinically relevant impact forces, or intensity levels that approximate those most likely to occur outside of a laboratory^57–61^. To replicate rapid, consecutive injuries, multiple impacts were administered at 4–6 s intervals, a reasonable estimate of the average time required to reset the apparatus between impact applications^25^.

#### Impact intervals: multiple-sequential impacts

The “Impact Interval”, or time separating multiple impacts, was increased from 4 to 6 s, to 120 min, and termed “multiple-sequential impacts”. Utilizing an impact force of 30 g, a series of three impacts were applied over a total period of 6 h. Networks resided in the chamber assembly in between impacts to eliminate unnecessary assembly/reassembly (and potentially stress). During this modified-incubation period, chamber assemblies were aseptically stored in an incubator to maintain temperature, and modified to allow free gas exchange for maintaining pH levels. It is important to note, that impact intervals of 120 min were carefully selected for their consistency with other models of TBI^62, 63^, and a particular focus on balancing injury intensity and “recovery-period” variables, two factors that have been shown to significantly influence experimental outcomes^64^. However, the system provides absolute command of both parameters, which can easily be adjusted to accommodate most needs.

#### Isolation of secondary injury

“Conditioned-media” (CM) is the term we use to describe media obtained from networks 4 h after they experienced maximum-limit impact forces (ten rapidly-imparted 200 g impacts), for the purpose of isolating and investigating secondary injuries. CM was obtained and substituted with media from non-impact networks for a total CM-exposure period of 24 h, followed by fixing.

Hydralazine (HZ, Hydralazine Hydrochloride, Sigma H1753), a repurposed antihypertensive drug and demonstrated acrolein scavenger that has already shown some promise in secondary injury models^65–67^, was introduced to CM and control networks at a concentration of 500 μM. Networks were exposed to CM for 15 min before HZ administration (CM + HZ), and incubated for 24 h before subsequent analysis.

#### The co-application of ethanol with TBI

To demonstrate the system’s ability to concurrently investigate relevant comorbidities, Ethanol (EtOH) was introduced post-injury at a concentration of 40 mM, a level comparable with mild intoxication in humans^68^ and below the EC50^69^. Utilizing a minor protocol modification, both single and multiple-rapid 30 g impacts (with separate controls) were administered, and immediately (~ 2 min) following the final impact application, media from control and impacted networks were replaced with a similar media containing EtOH for a duration of 15 min. After this exposure period ended, the EtOH-media was discarded and each culture’s original media was returned. All media was stored under sterile-incubation during any and all interim periods.

### Viability

As previously demonstrated, this method provides reliable, repeatable injuries in the absence of cell death^25^. However, for this investigation, a supplemental viability study was performed using fluorescein diacetate (FDA) and propidium iodide (PI), following standard procedures^70, 71^. Briefly, cells were thrice rinsed with isotonic phosphate buffered saline (PBS, pH = 7.4, Gibco), exposed to 25 μM and 7.5 μM of FDA and PI respectively for 5 min, and then imaged in PBS. Non-impact control networks resulted in an average viability of 97.5 ± 4.6%. When compared to networks exposed to 10 rapidly administered 30 g impacts, no significant difference was observed at 24 h post-impact (93.9 ± 6.1% viability, n = 5, p > 0.05). However, maximal impact forces of 10 rapidly administered 200 g impacts did reveal a significantly reduced average viability of 72.4 ± 8.4% (n = 5, p < 0.01).

### Immunofluorescence staining and antibodies

**Figure 2:**
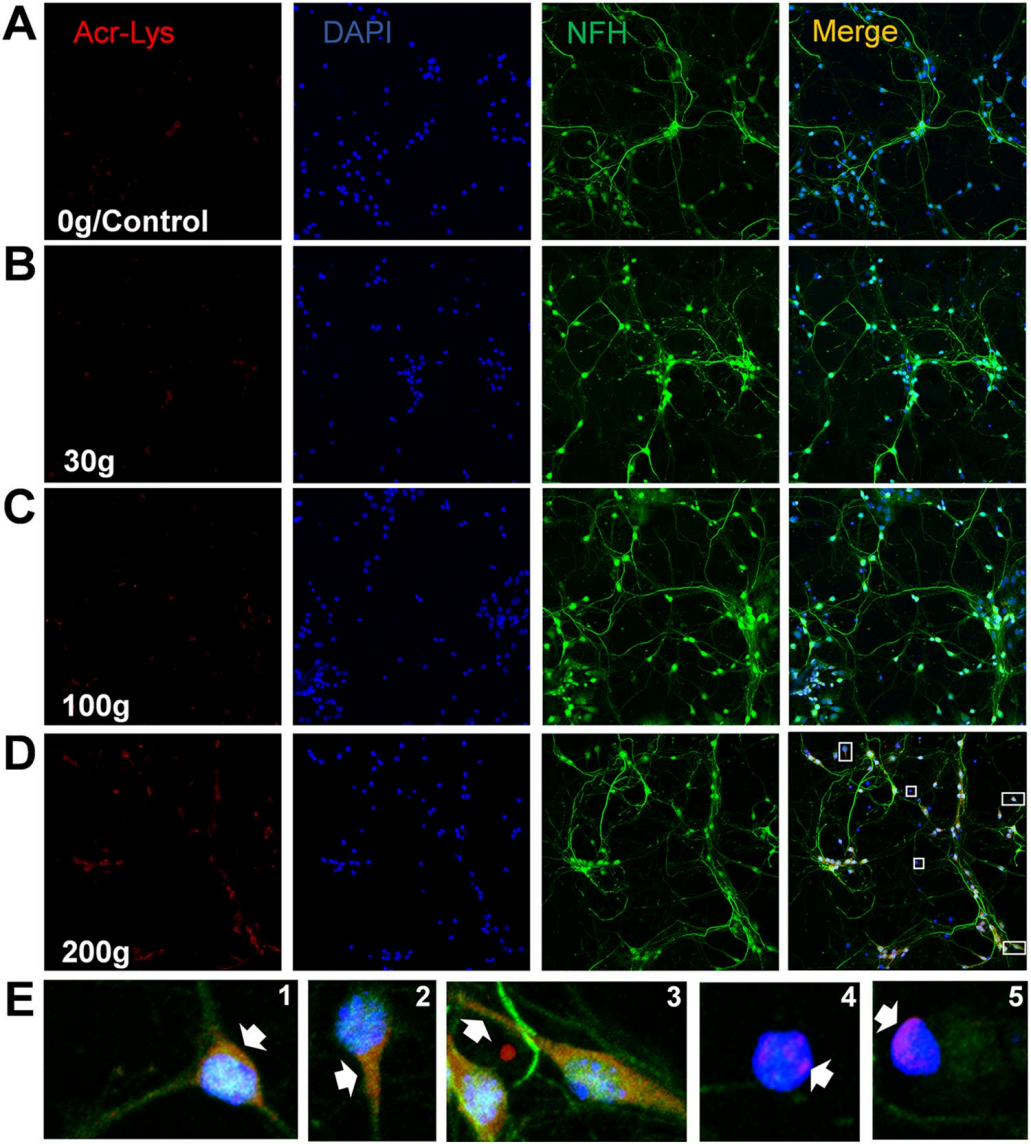
Visualizing the elevation of acrolein-Lys adducts as a function of single-impact severity. Representative fluorescence images at ×20 magnification are shown for histiotypical networks that received single impact forces of (**A**) 0 (control), (**B**) 30 g, (**C**) 100 g, (**D**) and 200 g. Nuclei are visualized with DAPI (blue), neuronal cell bodies and processes with neuron specific Neurofilament (green), and acrolein-Lys adducts with Stressmarq (red). Levels of acrolein-Lys adducts (red) progressively increase with larger g forces, moving top-to-bottom (**A**–**D**). Images demonstrating the spatial distribution of acrolein elevation are shown in both neuronal (E1-3) and non-neuronal cells (E4-5) which are enlarged from the (D)-merge series (200 g impact). Note the effective labeling of these antibodies, with the clear majority of all fluorescence being associated with cells, accompanied by very little background noise from our substrate and media. Further, these results suggest that all cells in our histiotypical cultures, both neuron (E1-3) and non-neuron (i.e. glia, E4&5), show increases in acrolein production, participating in the pathological consequences. However, most acrolein production appears to be perinuclear, with the majority localized in cell bodies (E1,4,5). While diffusion into cellular processes is observed (E2&3), it seems to be much less prevalent.

Neuronal network immunofluorescence was performed as previously described^72–74^, with minor modifications. All networks were thrice rinsed in PBS and fixed in 4% paraformaldehyde (Thermo Scientific) at 24 h post-treatment. Cultures were then washed with PBS, permeabilized with 0.2% Triton (Sigma), blocked with 10% normal donkey serum blocking solution (Abcam, AB7475), and treated overnight with primary antibodies at 4 °C. Subsequently, cells were gently rinsed with 0.1% Tween 20 (Sigma), treated with secondary antibodies for 2 h, again rinsed with tween, and bathed in PBS for imaging. This study utilized the following antibodies and stains: mouse anti-acrolein (Stressmarq, SMC-504D); Alexa Fluor 594 (Jackson Immunoresearch, 715-585-151); Alexa Fluor 488 (Jackson Immunoresearch, 703-545-155) Anti-NFH (Neurofilament Heavy Chain) (Aves Labs, NFH); and DAPI (Thermo Fisher, 62248). Quantitative measurements used relative light intensity of acrolein-Lys adduct levels taken with a customized Olympus IX81 fluorescence motorized phase contrast microscope and Qimaging EXi Blue CCD camera in MetaMorph Premier. Images analysis was performed with ImageJ. Representative fluorescence images (Fig. 2) were taken with a Zeiss LSM 880 Microscope with Airyscan (Carl Zeiss, Germany) using ZEN black software.

### Statistical analysis

Control and impacted network data for each treatment is reported from sets of 9 MEAs, each with 2 networks, and one average measurement per network (18 total, per treatment). All statistical analysis was performed using StataSE 16 and Microscoft Excel. Average values with standard deviations are used to show the variation in samples. Statistical significance between treatments was established with a one-way analysis of variance (ANOVA) and subsequent Scheffe post hoc analysis. Significance was defined as a p value < 0.05.

## Results

### The elevation of acrolein as a function of single and multiple impacts

**Figure 3:**
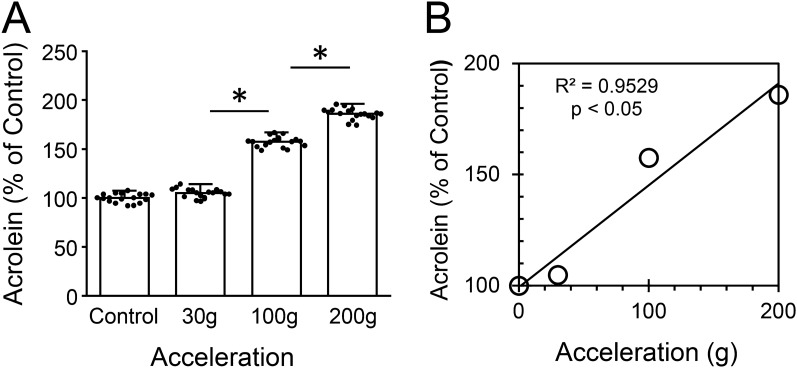
Quantifying the elevation of acrolein-Lys adducts as a function of single-impact severity. Immunocytochemistry (ICC) and immunofluorescence were used to detect and quantify acrolein-Lys adducts in histiotypical neuronal networks at 24 h post-impact, and compared with non-impacted, procedurally and age matched control-networks. (**A**) Average intensity measurements for Acrolein-Lys adducts given as percent control values for single impacts of 0 (control), 30, 100, and 200 g, (**B**) with the corresponding averaged acrolein values plotted as a function of acceleration to better illustrate this relationship (R^2^ = 0.95, p < 0.05). Staining intensity is shown as % control values ± SD (n = 18). One-way ANOVA and subsequent Scheffe post hoc analysis were used to determine significance: * p < 0.01.

Immunocytochemistry performed on networks experiencing impacts of 30, 100, and 200 g, as described in the “Methods” section, revealed significant, force-dependent increases of endogenous acrolein (acrolein-Lys adducts) production at 24 h post-impact (compared to age and procedurally matched control networks), and was visualized using fluorescent imaging (Fig. 2). High-resolution microscopy (Zeiss LSM 880, Airyscan) revealed predominantly perinuclear localization of acrolein elevation, with some occurrences in cellular processes. Results were quantified using immunostaining intensities for acrolein-Lys adducts, and are presented as average percent control values ± standard deviations, and increased as follows (Fig. 3): 4.9 ± 4.5% for mild impacts of 30 g (n = 18, p > 0.05, when compared to non-impact controls); 52.6 ± 5.3% for medium impacts of 100 g (n = 18, p < 0.01, when compared to 30 g); and 28.5 ± 5.6% for maximum impacts of 200 g (n = 18, p < 0.01, when compared to 100 g). Further, if the average increase in acrolein concentration is plotted against acceleration (Fig. 3B), it is evident that acrolein generation is a function of total g exposure.

**Figure 4:**
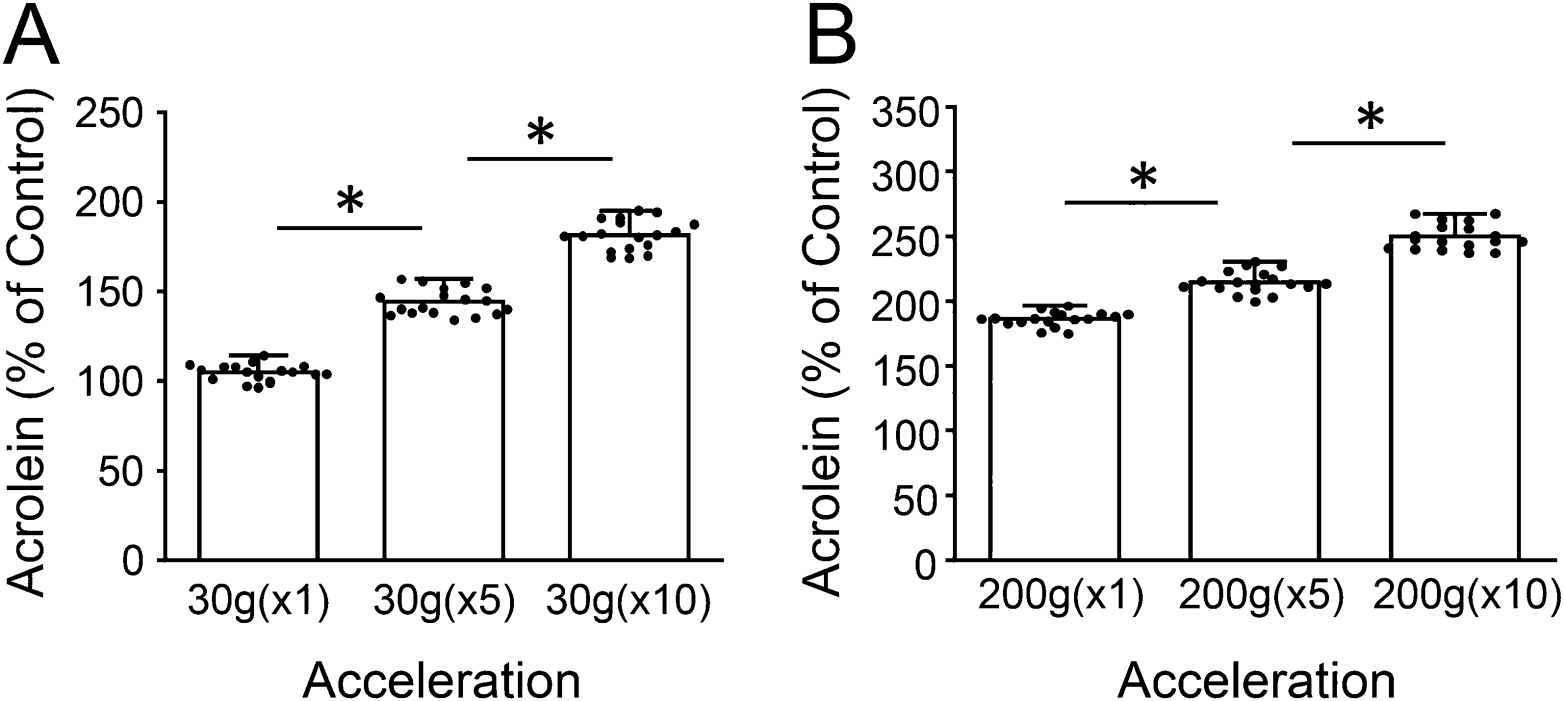
Elevation of acrolein-Lys adducts as a function of repeated impacts. Average intensity measurements for multiple impacts of 30 (**A**) and 200 g (**B**) rapidly (4–6 s) administered five and ten times. A single impact at both g forces levels is included for comparison. The increasing values displayed for both 30 and 200 g represent a cumulative effect of rapid, multiple impacts (note the difference in y-scale). Staining intensity is shown as percent control values ± SD (n = 18). One-way ANOVA and subsequent Scheffe post hoc analysis were used to determine significance: *p < 0.01.

In addition, multiple (5 and 10×) impacts applied in rapid (4–6 s) succession were also investigated (Fig. 4). Multiple-rapid impacts of 30 g resulted in acrolein-Lys adduct level increases. Again, when expressed as percent control, we observed average increases of 39.4 ± 7.5% for 30(×5) impacts (n = 18, p < 0.01, when compared to 30 g(×1) impacts), and 37.2 ± 8.6% for 30 g(×10) impacts (n = 18, p < 0.01, when compared to 30 g(×5) impacts) (Fig. 4A). Furthermore, rapidly administered impacts of 200 g revealed average increases of 28.5 ± 8.7% for 200 g(×5) impacts (n = 18, p < 0.01, when compared to 200 g(×1) impacts), and 35.4 ± 10.1% for 200 g(×10) impacts (n = 18, p < 0.01, when compared to 200 g(×5) impacts) (Fig. 4B). It is interesting to note, that while networks with single, mild impacts of 30 g did not reveal significant changes from controls, networks experiencing multiple-rapid 30 g impacts did. These results demonstrate a compounding effect, in addition to exhibiting the repeatability of the system.

### Elevation of acrolein as a function of impact interval duration

**Figure 5:**
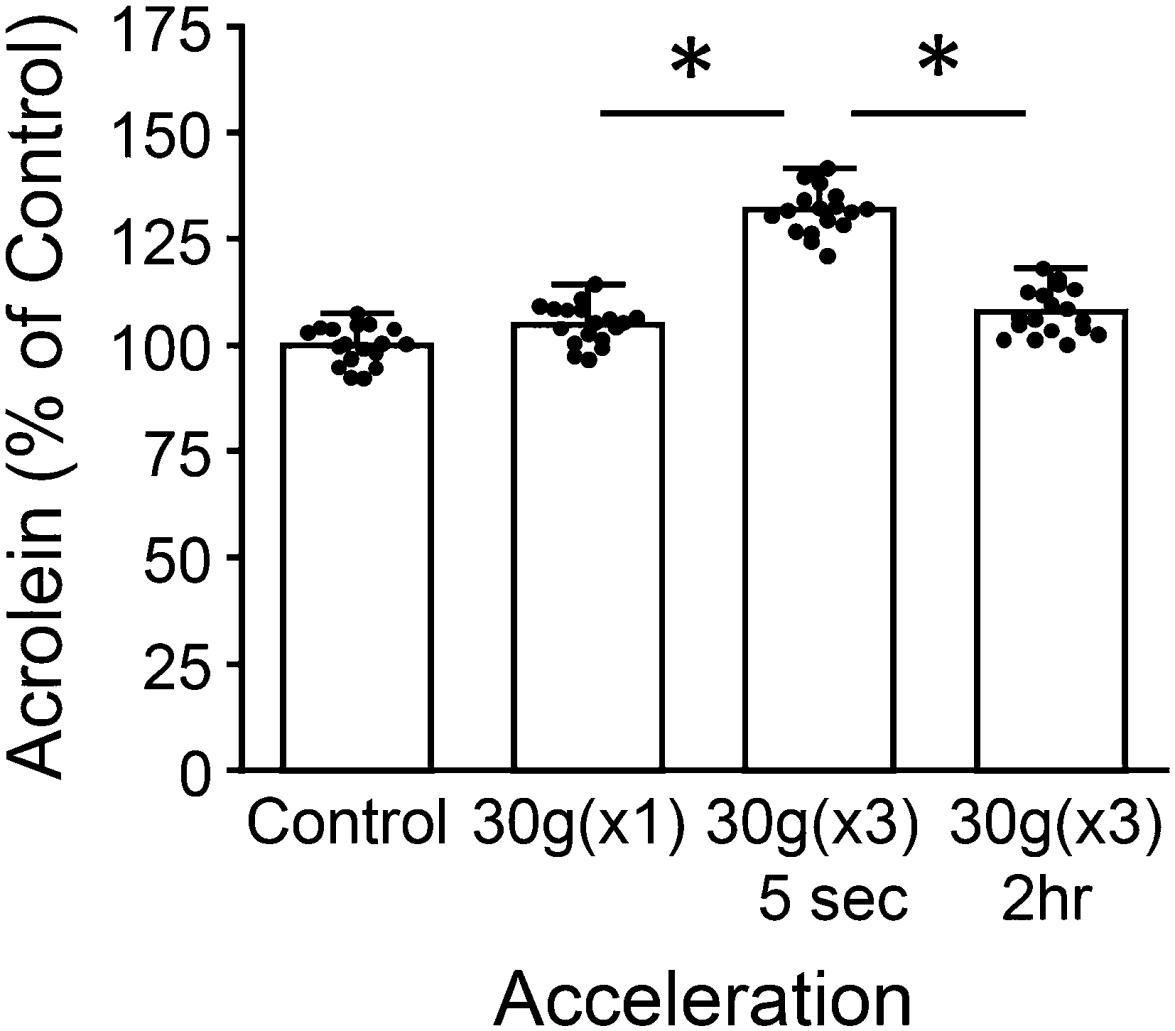
Increased inter-impact periods mitigate post-injury elevation of acrolein adducts. A 2 h time period was introduced between series of multiple impacts (multiple-sequential impacts), and average intensity measurements from ICC for acrolein-Lys adducts were performed at 24 h post-injury. Networks received three 30 g impacts in rapid succession (termed 30 g(×3)5 s) and were compared to networks that were exposed to three 30 g impacts separated by 2 h impact-intervals (termed 30 g(×3)2 h) and non-impact controls. A single 30 g impact-network is included for comparison. Interestingly, these inter impact intervals significantly decreased acrolein production when compared to 30 g(×3)5 s networks. Staining intensity is shown as percent control values ± SD (n = 18). One-way ANOVA and subsequent Scheffe post hoc analysis were used to determine significance: *p < 0.01.

Elongated impact intervals were introduced between multiple impacts, termed “multiple sequential impacts”, to investigate the role of inter-impact time in influencing post-injury acrolein elevation. Specifically, a 2-h impact interval was introduced between impact applications (Fig. 5). Three 30 g impacts were administered over a 6 h period (or three consecutive 2-h impact intervals, “30 g(×3) 2 h”), and fixed 24 h post final impact. As expected, the rapid application of three 30 g impacts, or “30 g(×3) 5 s”, increased acrolein-Lys levels on average by 27.1 ± 5.6% (n = 18, p < 0.01, when compared to 30 g(×1) impacts). However, the introduction of 2-h impact intervals reduced acrolein levels by 24.2 ± 5.4% (n = 18, p < 0.01) when compared to “30 g(×3) 5 s”.

### Conditioned media from impacted cultures causes acrolein elevation in non-impacted cells

**Figure 6:**
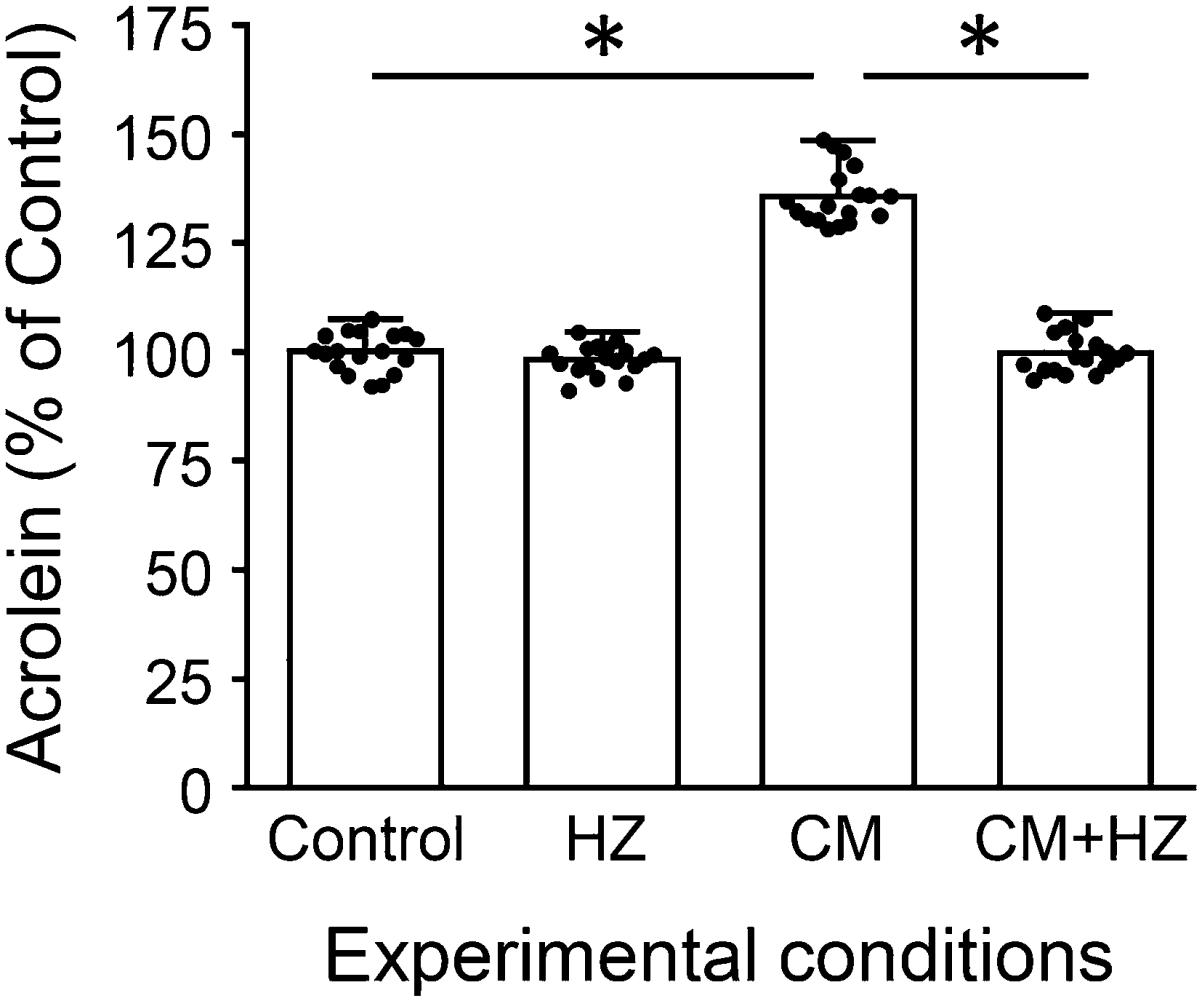
Isolating acrolein in post-impact diffusive secondary injuries. Culture medium from networks that experienced relatively large impact forces (200 g impacts rapidly administered 10x) was substituted in non-impact networks and incubated for 24 h. After incubation, ICC and subsequent intensity measurements were performed to evaluate acrolein-Lys adduct levels. This now “conditioned-medium,” or CM, allows for the separation of secondary from primary traumatic injury by removing the direct application of mechanical force from the procedure. Interestingly, CM network’s (non-impact networks exposed to treated media) average acrolein levels rose significantly. Further, the acrolein scavenger hydralazine was then introduced post conditioned media exposure at a concentration of 500 µM (CM + HZ). This resulted in a significant decrease of average acrolein production when compared with CM networks. Further, when these values were compared with non-impact controls (Control) and networks given identical concentrations of hydralazine with standard, non-conditioned medium (HZ), no significant changes were found. Staining intensity is shown as percent control values ± SD (n = 18). One-way ANOVA and subsequent Scheffe post hoc analysis were used to determine significance: *p < 0.01.

To investigate the model’s potential for isolating media-tied secondary injuries from mechanically linked-primary injuries, cell culture media from non-impacted networks was replaced with “conditioned-medium” from maximally impacted networks (CM), as described in the “Methods” section. After a 24 h exposure period, acrolein-Lys adduct levels in non-impacted CM-networks rose by an average of 35.7 ± 6.5% (n = 18, p < 0.01, when compared to control networks) (Fig. 6). In addition, the acrolein scavenger hydralazine was introduced to conditioned media experiments (CM + HZ) at a concentration of 500 μM with a matching positive control (HZ). Hydralazine addition significantly reduced acrolein levels in CM + HZ networks by 36.0 ± 4.6% (n = 18, p < 0.01, as compared to CM networks), resulting in values with no significant difference from control networks (p > 0.05).

### Ethanol exacerbates acrolein elevation post-impact

**Figure 7:**
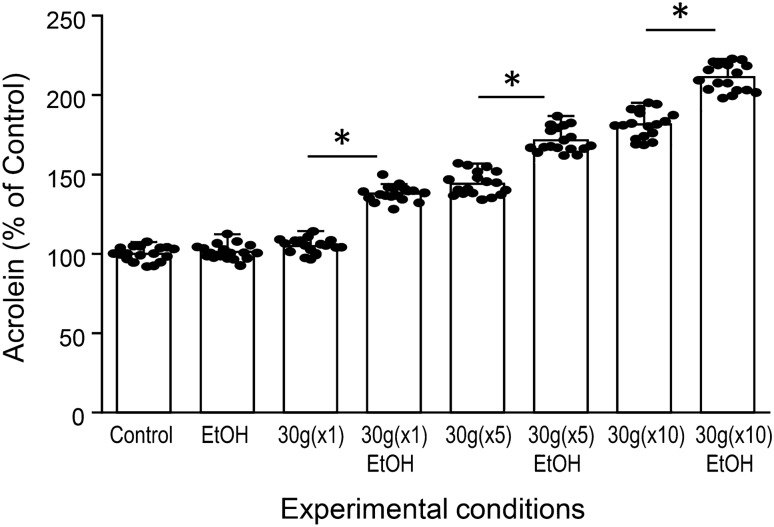
Ethanol exposure post-impact exacerbates acrolein elevation. Ethanol was introduced to demonstrate the system’s capability to easily investigate potential comorbidities. Networks experiencing rapid (5 s) 30 g impacts in single, ×5, or ×10 quantities were exposed to 40 mM EtOH for a duration of 15 min immediately (~ 2 min) following injury (30 g(× 1)EtOH, 30 g(×5)EtOH, and 30 g(×10)EtOH, respectively) and compared with identical, EtOH-absent treatments (30 g(×1), 30 g(×5), and 30 g(×10), respectively). ICC for acrolein-Lys adducts was performed at 24 h post-injury. EtOH significantly increased acrolein levels compared to non-EtOH exposed impact-networks of force and quantity matched impact forces in all cases. Non-impact control networks with and without EtOH (Control and EtOH, respectively) showed no significant differences. Staining intensity is shown as percent control values ± SD (n = 18). One-way ANOVA and subsequent Scheffe post hoc analysis were used to determine significance: *p < 0.01.

Ethanol (EtOH) was introduced to networks experiencing both single and multiple (×1, ×5, and ×10) mild 30 g impacts. 40 mM EtOH exposure for a duration of 15 min post-impact significantly increased acrolein-Lys adduct levels at 24 h when compared with ethanol-free impacts of matching forces (Fig. 7). When compared to non-EtOH exposed impact-force and quantity matched networks, EtOH exposure increased average acrolein production by 33.1 ± 4.9% (n = 18, p < 0.01), 27.4 ± 7.7% (n = 18, p < 0.01), and 29.9 ± 8.6% (n = 18, p < 0.01), for ×1, ×5, and ×10 impacts of 30 g, respectively. Non-impacted EtOH exposed networks did not show significant changes in acrolein levels when compared to control networks (p > 0.05).

## Discussion

Using a recently developed TBI-on-a-chip in vitro model, in which precise forces can be systematically applied to mimic concussion injury^25^, we have demonstrated that a single impact within a clinically relevant range of g forces (30–200 g)^60, 61^, results in a measurable elevation of acrolein, a toxic aldehyde and a known marker of CNS pathological injury^12–14, 18, 24, 26, 27, 32, 41, 75^, at 24 h post-impact in a force-dependent fashion. In addition, we have also shown that, while single impacts of 30 g did not result in statistically significant increases of acrolein after 24 h, the rapid (4–6 s) administration of multiple (3,5, and 10) 30 g impacts significantly amplified these acrolein elevations in proportion to the total number of hits. These changes are similar to the cumulative damage demonstrated by in vivo models of repetitive-TBI^76, 77^. Further, these repetitive-impact induced elevations in acrolein can be significantly reduced, resulting in levels comparable to networks that only experienced a single impact, by increasing the inter-impact intervals to 2 h, recapitulating a phenomenon seen in multiple in vivo TBI studies and hypothesized to reflect a recovery mechanism^62, 78^. Also with this model, we show that endogenously produced acrolein, known to be capable of diffusing away from injured neuronal cells^13, 26, 66^, can reach extracellular concentrations high enough to induce further acrolein elevation in un-impacted, and otherwise healthy cells. We have also found that while the majority of impact-induced acrolein elevation is confined to cell bodies, some detection in cellular processes was also noted. This observation is supported by our previous in vivo/ex vivo studies, which revealed increases in trauma-elicited acrolein in both grey and white matter^26, 66^, which is also correlated with membrane damage and oxidative stress following mechanical insult^79, 80^. Similarly, several studies utilizing animal models of trauma have specifically isolated acrolein as a significant, diffusive factor in secondary injury, capable of exacerbating and even propagating damage in CNS trauma^12, 26, 42, 52, 66, 81^. Taken together, the current study provides supporting evidence for these claims, further suggesting that acrolein plays a key role in secondary injury, particularly during the acute stages, while also demonstrating the effectiveness of the model^12, 24, 48, 82, 83^. However, it is important to note that while the current, relevant literature has reported acrolein levels up to 14 days post-injury^52^, longer-term studies will ultimately be required for a more comprehensive understanding of acrolein’s precise role in chronic-injury. Finally, we have confirmed that clinically relevant concentrations of ethanol (40 mM) at limited exposure periods (15 min)^68^, a treatment that did not cause acrolein elevation when administered alone to un-impacted neuronal cells, can further-significantly elevate acrolein when introduced following single or repeated injuries, even at minimal force intensities (30 g). These results suggest a synergistic effect of ethanol in exacerbating oxidative stress when combined with mechanical injury. This is expected to open the door for further investigations into the underlying mechanisms and pathological consequences of TBI that are accompanied by alcohol consumption, as well as many other clinically relevant comorbidities.

Taken together, the current TBI-on-a-chip preparation signifies a novel, in vitro model for simulating concussion injury. Furthermore, it has also demonstrated the ability to isolate secondary biochemical-injury mechanisms (i.e., acrolein), incorporate comorbidities (i.e., alcohol), and serve as a semi-high throughput preparation for investigating potential pharmaceutical interventions (i.e., the acrolein scavenger hydralazine). Combining the ability to concurrently assess functional alterations in the brain by analyzing changes in neuronal network activity (as demonstrated in a previous report^25^), we feel that the present model represents a new and versatile in vitro system capable of rigorously investigating the mechanisms of injury by correlating mechanical, biochemical, and electrophysiological assessments, all in the same preparation. By mimicking clinically relevant mechanical insults, the device can not only recapitulate key pathological features seen in vivo post-TBI, but also with higher resolution in cellular and sub-cellular levels, offering a powerful complementary tool for in vivo studies and multidisciplinary-investigations. The knowledge gained from the current model is expected to provide insight into the mechanisms of TBI while also suggesting more effective biomarkers that could lead to earlier diagnoses, as well as introduce more effective treatments.

While other pathologically relevant models, that utilize neuronal cell cultures with similar temporal and spatial resolution, have been established^63^, few have been able to accurately recapitulate the physical, mechanical impact seen with concussive injuries^25, 84, 85^. This is due in part to the associated technical difficulties somewhat inherent to these relatively fragile, in vitro culture systems, and thus proper cell-substrate adhesion becomes paramount. We have overcome these obstacles using a tangential impulse (impact) provided by a ballistic pendulum, which laterally imparts impact forces to cells safely contained in a custom-engineered “impact-chamber” (Fig. 1). This results in consistent, relatively-clean biomechanical acceleration forces that stress cellular components and subcellular mechanisms, while maintaining neuronal adhesion on the MEA substrate^25^.

Utilizing the current model, we also noted acrolein’s subcellular spatial distribution, an observation which may provide insight into the origin of these acrolein surges following mechanical stress. It is well known that acrolein can be endogenously produced through lipoperoxidation (LPO)^86^, a process that can result from oxidative stress which, again, can be elicited via force-induced membrane damage^13, 14, 87–90^. Membrane damage is one of the initial, cellular pathological events that has been shown to occur following a physical insult, a phenomenon that has been well documented in CNS trauma^79, 91–98^. As such, it is possible that the acute elevations of acrolein demonstrated in these cultured cells are initiated by a physical injury to the membrane. As shown (Fig. 2), the majority of acrolein production was confined to cell bodies, suggesting that the soma-associated plasma membrane is the main site of initial damage in this model. This finding is somewhat intuitive, as the increased inertia of the now impacted nucleus, the largest organelle in these cells, would exacerbate local membrane damage^99^.

It is interesting to note that while the majority of acrolein production occurs in the soma, acrolein has also been observed in cellular processes: both somewhat limited in the proximal segment, and other times seeming to span the entire process-length. Whether these spatial-alterations in acrolein-elevation is caused directly via process damage or by diffusing away from the cell body is not clear. However, this and previous studies support the hypothesis that perinuclear acrolein diffuses into cellular processes. First, we have shown that acrolein can spread from the site of injury in white matter towards neighboring axonal segments in an animal model of spinal cord injury^26^. Second, in the current study we observed that acrolein elevation always occurs in the cell body, but is only rarely accompanied by the presence of acrolein in processes. In other words, process-specific acrolein elevation was never witnessed alone. While this finding seems to reinforce our diffusion-hypothesis, it does not rule out the possibility of increased acrolein generation in processes directly, as membrane damage could occur at both locations. Regardless, because membrane damage is likely the main reason for early acrolein elevation in both cases, membrane repair could offer a viable strategy to reduce acrolein production. Therefore, the effectiveness of acrolein scavengers such as hydralazine may be further aided by the addition of a membrane repairing agent, like Polyethylene Glycol (PEG)^93, 94, 96–98, 100^. This synergistic approach could sequester the acrolein that is already present while simultaneously working to minimize damage and further production of acrolein, and can be readily investigated using the current TBI-on-a-chip-model.

It is becoming increasingly evident that the most severe TBI-induced damage does not occur immediately following the physical impact, which can be particularly pronounced in cases of mild brain injuries (mTBI)^101–104^. Rather, the initial mechanical insult induces a cascade of biochemical reactions that amplify the primary effects and proliferate damage throughout the brain, leading to a delayed secondary neurological injury. Therefore, while investigations into primary injury mechanisms are important, the concurrent ability to elicit, monitor, and intervene into known secondary injuries that have been previously verified in vivo, is a critical component of a viable and relevant in vitro TBI model. TBI-on-a-chip utilizes mechanical impacts to recapitulate acrolein elevation, offering a unique opportunity to assess primary injury and the pathological mechanisms of acrolein-related damages. This includes providing an option to separate media-tied biochemical secondary injuries using our “Conditioned Media” protocol and the opportunity to investigate the effects of potential pharmaceutical interventions like the acrolein scavenger hydralazine (a well-established neuroprotective strategy in preclinical in vivo studies)^52, 66^.

In the current study, all acrolein measurements were performed at 24 h after injury. However, TBI-on-a-chip’s in vitro design enables the investigation of much earlier time points, allowing for the observation of acrolein changes (or other molecules of interest) in mere minutes after impact and offering information into aldehyde dynamics with temporal and spatial resolution not attainable in any other concussive model. For example, in the current study we find that a single 30 g impact did not result in a significant elevation of acrolein levels at 24 h post-impact. However, it is possible that more transient increases of acrolein occur in the minutes or hours post-injury, a difficult hypothesis to examine in vivo, but one that can be readily tested using the current in vitro model. This could potentially offer valuable insight into the dynamics of post-TBI pathology, facilitating earlier stage diagnoses and treatments.

This study confirms our model’s ability to accurately replicate the mechanical insults (primary injury) and subsequent biochemical cascades (secondary injury) associated with trauma. This suggests that the current model will also offer an effective preparation for screening pharmacological agents and their propensity for both repairing primary injury, such as PEG^93^, and also suppressing secondary injuries, such as acrolein scavengers^18, 49^. Although multiple acrolein scavengers, including hydralazine, have been shown to sequester acrolein in various in vivo mechanical injury models of CNS trauma^49, 50, 52, 66, 105^, this is the first time that hydralazine-mediated acrolein reduction has been shown in an in vitro mechanical TBI model. This reflects both the significance of the TBI-on-a-chip model in investigating injury mechanisms, as well as its utility to facilitate drug discovery efforts aimed at establishing effective pharmacological interventions to offer neuroprotective benefits.

The recreational consumption of alcohol is not only a readily observed social phenomenon, but has been scientifically demonstrated in multiple population studies of groups with a higher propensity for TBI, including athletes and military personnel, both active duty and reserve^106^. It has been suggested that such consumption behaviors can even significantly increase after TBI, a currently under recognized factor which could potentially impair long-term recovery. Although multiple studies have investigated the effects of prior, chronic alcohol-usage on TBI, few have examined the pathological consequences of alcohol consumption post-TBI. In the current study, neither a single impact of minimum intensity (30 g) or a brief exposure (15 min) of 40 mM ethanol resulted in significant increases of acrolein production. However, when these two treatments were combined, they surpassed the threshold of significance. This is interesting, as other studies have suggested ethanol’s both neuroprotective^107–110^ and toxic^111–113^ properties when combined with traumatic injury, implying that exposure time, duration, and concentration may determine the outcome of such interplay. While acrolein-mediated downstream pathological changes were not conducted in this initial study, there is little doubt that this degree of acrolein elevation will likely result in multiple molecular neuronal pathologies, including oxidative stress and inflammation. However, this study demonstrates that, at these concentrations in vitro, ethanol alone is not capable of significantly modulating oxidative stress levels as measured via acrolein production, until incorporation with traumatic events. Other mechanisms are likely involved, and are the focus of an upcoming study. Future studies using the current model could also potentially reveal secondary molecular mechanisms, in parallel or downstream, after acrolein elevation, which could serve as therapeutic targets for neuroprotection.

## Data Availability

Data files are available for all experiments upon request.
